# Effect of omega-3 fatty acid supplementation on cancer incidence, non-vascular death, and total mortality: a meta-analysis of randomized controlled trials

**DOI:** 10.1186/1471-2458-14-204

**Published:** 2014-02-26

**Authors:** Yu-Fei Zhang, Hong-Fang Gao, An-Ji Hou, Yu-Hao Zhou

**Affiliations:** 1 Department of Oncology, Shanghai Seventh People’s Hospital, Shanghai, China; 2 Department of Rehabilitation Institute, Shanghai Seventh People’s Hospital, Shanghai 200137, China

**Keywords:** Omega-3 fatty acid, Cancer, Mortality, Meta-analysis

## Abstract

**Background:**

Omega-3 fatty acids are known to prevent cardiac death. However, previous observational studies have suggested that omega-3 fatty acids are associated with cancer risk in adults. We conducted a meta-analysis based on randomized controlled trials to evaluate the effect of omega-3 fatty acids on the risk of cancer incidence, nonvascular death, and total mortality.

**Methods:**

In February 2013, we performed electronic searches in PubMed, EmBase, and the Cochrane Library to identify randomized controlled trials on cancer incidence, nonvascular death, and total mortality. Relative risk (RR) was used to measure the effect of omega-3 fatty acid supplementation on the risk of cancer incidence, nonvascular death, and total mortality using a random-effect model. The analysis was further stratified by factors that could affect the treatment effects.

**Results:**

Of the 8,746 identified articles, we included 19 trials reporting data on 68,954 individuals. These studies reported 1,039 events of cancer, 2,439 events of nonvascular death, and 7,025 events of total mortality. Omega-3 fatty acid supplementation had no effect on cancer incidence (RR, 1.10; 95% CI: 0.97–1.24; P = 0.12), nonvascular death (RR, 1.00; 95% CI: 0.93–1.08; P = 1.00), or total mortality (RR, 0.95; 95% CI: 0.88–1.03; P = 0.24) when compared to a placebo. Subgroup analysis indicated that omega-3 fatty acid supplementation was associated with a reduction in total mortality risk if the proportion of men in the study population was more than 80%, or participants received alpha-linolenic acid.

**Conclusions:**

Omega-3 fatty acid supplementation does not have an effect on cancer incidence, nonvascular death, or total mortality.

## Background

Omega-3 fatty acid supplementation has been suggested to reduce the risk of cancer incidence [1-3], including that of colorectal [1], lung [2], and prostate [3] cancers. However, observational studies often overestimate the size of the effect and do not prove causality. Omega-3 fatty acid-derived eicosanoids influence many physiological processes such as calcium transport across cell membranes, angiogenesis, apoptosis, cell proliferation, and immune cell function [4-6], all of which might play a role in cancer risk.

Omega-3 fatty acid supplementation has been studied in numerous, large-scale, randomized, controlled trials for primary and secondary prevention of cardiovascular outcomes [7-17]. We could gain insight into the risk of cancer between omega-3 fatty acid supplementation and a placebo, with a study involving a long-term follow-up period and proper collection of cancer data. Therefore, we conducted a systematic review and meta-analysis of pooled data from randomized controlled trials to evaluate the possible effect of omega-3 fatty acid supplementation on cancer incidence, nonvascular death, and total mortality.

## Methods

### Data sources, search strategy, and selection criteria

This review was conducted and reported according to the Preferred Reporting Items for Systematic Reviews and Meta-Analysis (PRISMA) Statement [18], issued in 2009 (Additional file 1: Table S1). Randomized controlled trials of omega-3 fatty acid supplementation, written in the English language, were eligible for inclusion in our meta-analysis, regardless of the publication status (published, in press, or in progress), and the effects of omega-3 fatty acid supplementation on cancer incidence, nonvascular death, and total mortality were examined. Relevant trials were identified using the following procedure:

(1) Electronic searches: we searched the PubMed, EmBase, and Cochrane Central Register of Controlled Trials electronic databases for articles published through February 2013 and used “linolenic acid” OR “timnodonic acid” OR “ALA” OR “EPA” OR “docosahexaenoic acid” OR “DHA” OR “omega-3 fatty acid” OR “fish oil” AND “randomized controlled trials” AND “clinical trials” AND “human” as the search terms. All reference lists from reports on non-randomized controlled trials were searched manually for additional eligible studies.

(2) Other sources: we searched ongoing randomized controlled trials in the metaRegister of Controlled Trials, which lists trials that are registered as completed but not yet published. Furthermore, we reviewed bibliographies of publications for potentially relevant trials. Medical subject headings, methods, patient population, interventions, and outcomes variables of these studies were used to identify relevant trials.

The literature search, data extraction, and quality assessment were undertaken by two authors (YFZ and AJH), independently with a standardized approach. Any inconsistencies between these 2 authors (YFZ and AJH) were settled by the primary author (YHZ) until a consensus was reached. We restricted our research to randomized controlled trials, which were less likely to be subjected to confounding variables or bias views than observational studies. The study was eligible for inclusion if the following criteria were met: (1) the study was a randomized controlled trial; (2) the trial evaluated the effects of omega-3 fatty acid supplementation compared to a placebo; (3) the duration of the study’s follow-up period was at least 6 months; and (4) the trial reported at least 1 outcome as either cancer incidence, nonvascular death, or total mortality.

### Data collection and quality assessment

All data from eligible trials were independently abstracted, in duplicate, by 2 independent investigators (YFZ and HFG) with a standard protocol and reviewed by a third investigator (YHZ). Any discrepancies were resolved by a group discussion. The data collected included baseline characteristics (first author or study group’s name, publication year, study design, type of blinding, number of patients, mean age, percentage of males, patient diseases, intervention regimes, and the duration of the follow-up period). The outcomes investigated included cancer incidence, nonvascular death, and total mortality. Study quality was assessed using the Jadad score [19], which is based on the 5 following subscales: randomization (1 or 0), concealment of the treatment allocation (1 or 0), blinding (1 or 0), completeness of follow-up (1 or 0), and the use of intention-to-treat analysis (1 or 0). A “score system” (ranging from 1 to 5) has been developed for quality assessment. In our study, we considered a study given a score of 4 or above to be a high-quality study.

### Statistical analysis

We allocated the results of each randomized controlled trial as dichotomous frequency data. Individual study relative risks (RRs) and 95% confidence intervals (CIs) were calculated from event numbers extracted from each trial before data pooling. The overall RR and 95% CIs of cancer incidence, nonvascular death, and total mortality were also calculated. Both fixed-effect and random-effects models were used to assess the pooled RR for omega-3 fatty acid supplementation compared to a placebo. Although both models yielded similar findings, results from the random-effects model presented here assume that the true underlying effect varies among included trials [20,21]. Heterogeneity of the treatment effects between studies was investigated visually by a scatter plot analysis as well as statistically using the heterogeneity I^2^ statistic [22,23]. We explored potential heterogeneity in estimates of the treatment effects with univariate meta-regression (for sample size, mean age, percent men, and duration of the follow-up period) [24]. Subsequently, subgroup analyses were conducted on the basis of publication year, number of patients, percent men, mean age, intervention, primary or secondary prevention, duration of the follow-up period, and the Jadad score [19]. Interaction tests [25] were performed to compare differences between estimates of the 2 subsets, which were based on Student *t* distribution rather than on normal distribution because the number of inclusive studies was small. We also performed a sensitivity analysis by removing each individual trial from the meta-analysis. Visual inspections of funnel plots for cancer incidence, nonvascular death, and total mortality were conducted. The Egger [26] and Begg tests [27] were also used to statistically assess publication bias for cancer incidence, nonvascular death, and total mortality. All reported P values are two-sided, and P values of <0.05 were considered statistically significant for all included studies. Statistical analyses were performed using STATA software (version 10.0).

## Results

**Figure 1:**
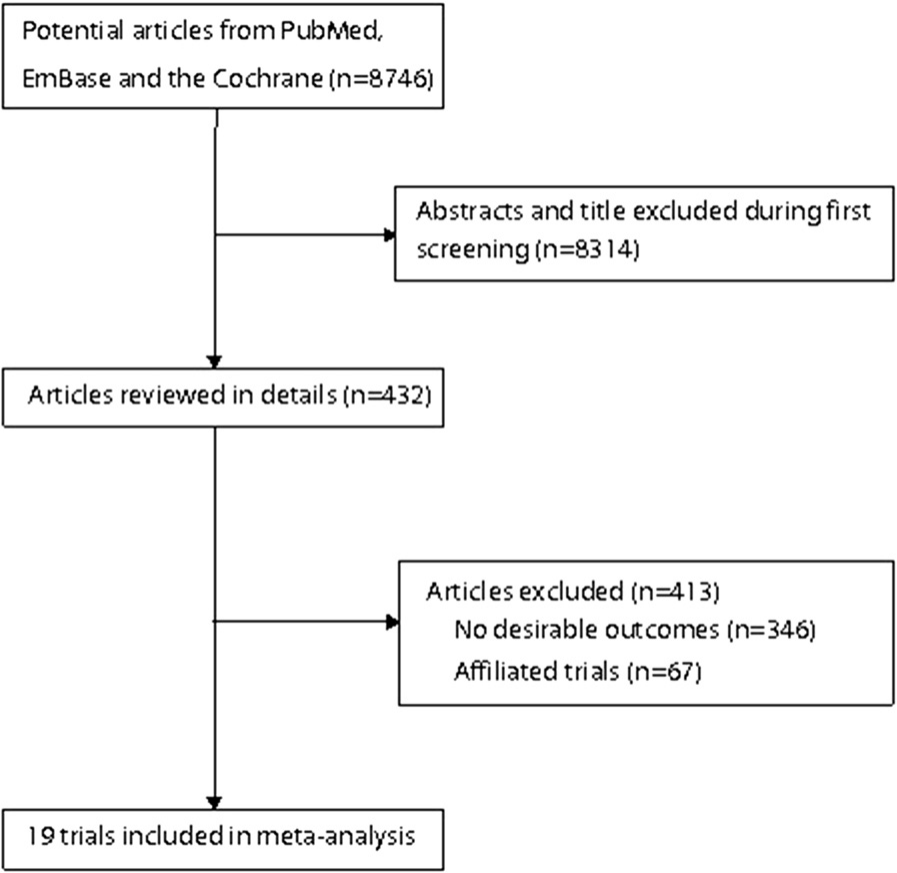
Flow diagram of the literature search and trials selection process.

We identified 8,746 articles from our initial electronic search, of which 8,314 were excluded during an initial review (title and abstract). We retrieved the full text for the remaining 432 articles, and 19 randomized controlled trials [7-17,28-35] met the inclusion criteria (Figure 1 and Additional file 2: Figure S1). Table 1 summarizes the characteristics of these trials and the important baseline information of the included 68,954 individuals. The trials included in this study compared omega-3 fatty acid supplementation to a placebo for cancer incidence, nonvascular death, and total mortality. The follow-up period for participants ranged from 10 to 74.4 months, and the number of individuals included in each trial ranged from 36 to 18645. The breakdown for the number of trials available for each outcome were 11, 16, and 17 for cancer incidence [7-17], nonvascular death [7,8,11-16,28-35], and total mortality [7,8,11-17,28-35], respectively. We restricted the inclusion criteria to randomized controlled trials with a 6-month minimum follow-up to ensure a reliable conclusion. Although the included trials scarcely reported on the key indicators of trial quality, the quality of the trials was also assessed by the Jadad score [19]. Overall, 4 trials had a Jadad score of 5, 7 trials had a score of 4, 4 trials had a score of 3, 2 trials had a score of 2, and the remaining 2 trials had a score of 1.

**Table 1:** Design and baseline characteristic of trials included in the systematic review and meta-analysis

**Source**	**No. of patients**	**Mean age, y**	**Sex (male, %)**	**Disease status**	**Intervention**	**Control**	**Follow-up (months)**	**Reporting outcomes**	**Jaded score**
Borchgrevink CF (1966) [7]	100/100	57.4	100	Coronary heart disease	10 ml linseed oil	10 ml corn oil	10	Cancer incidence; nonvascular death; total death	2
Burr ML (1989) [8]	1015/1018	56.5	100	Recovered from myocardial infarction	Fish dietary	Non-fish dietary	24	Cancer incidence; Nonvascular death; total death	1
de Lorgeril M (1994) [28]	302/303	53.5	90.8	Myocardial infarction within 6 months	Alpha-linolenic acid rich diet	Post-infarct prudent diet	27	Nonvascular death; total death	1
Rossing P (1996) [9]	18/18	33.0	65.5	Diabetic nephropathy	N–3 fatty acids	Olive oil	12	Cancer incidence;	4
Leng GC (1998) [29]	60/60	65.7	68.3	Stable intermittent claudication	Gamma-linolenic and eicosapentaenoic acids	Placebo	24	Nonvascular death; total death	3
von Schacky (1999) [10]	111/112	58.4	80.3	Coronary artery disease	Fish oil concentrate (55% eicosapentaenoic and docosahexaenoic acids)	Placebo	24	Cancer incidence;	5
GISSI-Prevenzione Investigators (1999) [11]	5666/5658	60.0	85.3	Recovered from myocardial infarction	N-3 polyunsaturated fatty acids witho or without vitamin E	Vitamin E or no treatment	42	Cancer incidence; Nonvascular death; total death	3
Nilsen DWT (2001) [12]	150/150	64.0	79.4	Acute myocardial infarction	4 g highly concentrated n-3 fatty acids	Corn oil	24	Cancer incidence; nonvascular death; total death	2
Burr ML (2003) [13]	1571/1543	61.1	100	Angina	Two portions of oily fish each week, or to take three fish oil capsule daily with or without more fruit, vegetables and oats	no specific dietary advice with or without more fruit, vegetables and oats	60	Cancer incidence; nonvascular death; total death	3
Raitt MH (2005) [14]	100/100	62.5	86	Implantable cardioverter defibrillator and a recent episode of sustained VT or VF	Fish oil, 1.8 g/d, 72% omega-3 PUFAs	Placebo	24	Cancer incidence; nonvascular death; total death	4
Leaf A (2005) [30]	200/202	65.5	83.1	Implantable cardioverter defibrillator	Fish oil	Olive oil	12	Nonvascular death; total death	3
Brouwer IA (2006) [15]	273/273	61.5	84.5	Implantable cardioverter defibrillator	2 g/d of fish oil	Placebo	12	Cancer incidence; nonvascular death; total death	4
JELIS Ivestigators (2007) [16]	9326/9319	61.0	31.5	Total cholesterol of 6 · 5 mmol/L or greater	EPA with statin	Statin	55.2	Cancer incidence; nonvascular death; total death	5
GISSI-HF investigators (2008) [31]	3494/3481	67.0	NG	Chronic heart failure	N-3 PUFA 1 g daily	Placebo	46.8	Nonvascular death; total death	5
OMEGA Study Group (2010) [32]	1919/1885	64.0	74.4	Recovered from myocardial infarction	1 g omega-3 acid ethyl esters-90	Olive oil	12	Nonvascular death; total death	4
Einvik G (2010) [33]	282/281	70.1	100	Hypercholesterolemia (> 6.45 mmol/l)	2.4 g n-3 PUFA	Corn oil	36	Nonvascular death; total death	4
SU.FOL.OM3 Collaborative Group (2010) [17]	1253/1248	60.6	79.4	with a history of myocardial infarction, unstable angina, or ischaemic stroke	Omega 3 fatty acids with or without vitamin B	Placebo or vitamin B	56.4	Cancer incidence; total death	5
Alpha Omega Trial Group (2010) [34]	2404/2433	69.0	78.2	Recovered from myocardial infarction	EPA and DHA with or without ALA	ALA or placebo	40.8	Nonvascular death; total death	4
The ORIGIN Trial Investigators (2012) [35]	6281/6255	63.5	65.1	High risk for cardiovascular events	N–3 fatty acids	Placebo	74.4	Nonvascular death; total death	4

**Figure 2:**
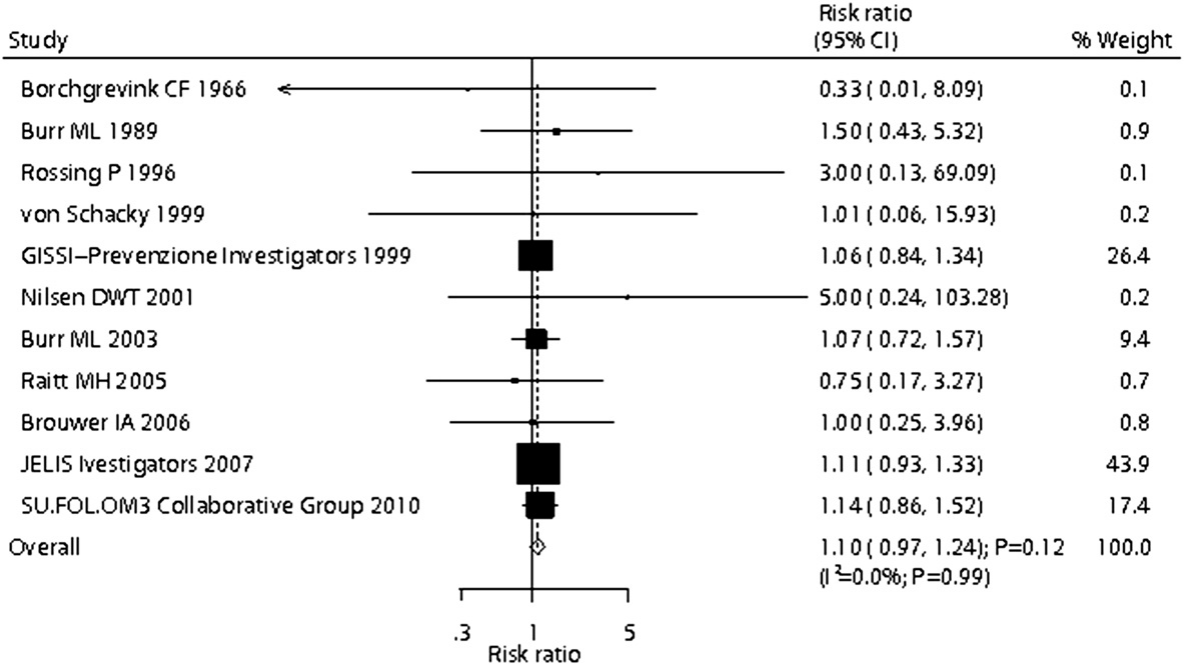
Effect of omega-3 fatty acid supplementation on the risk of cancer incidence.

Data for the effect of omega-3 fatty acid supplementation on cancer incidence included 39,122 individuals and reported 1,039 cancer events. Overall, omega-3 fatty acid supplementation increased the risk of cancer incidence by 10%, but this increase was not statistically significant (RR, 1.10; 95% CI: 0.97–1.24; P = 0.12, without evidence of heterogeneity, Figure 2). A sensitivity analysis indicated that the results were not affected by sequential exclusion of any particular trial from all pooled analysis. Similarly, the non-significant response persisted after a cumulative meta-analysis for cancer incidence was conducted (Additional file 3: Figure S2).

**Figure 3:**
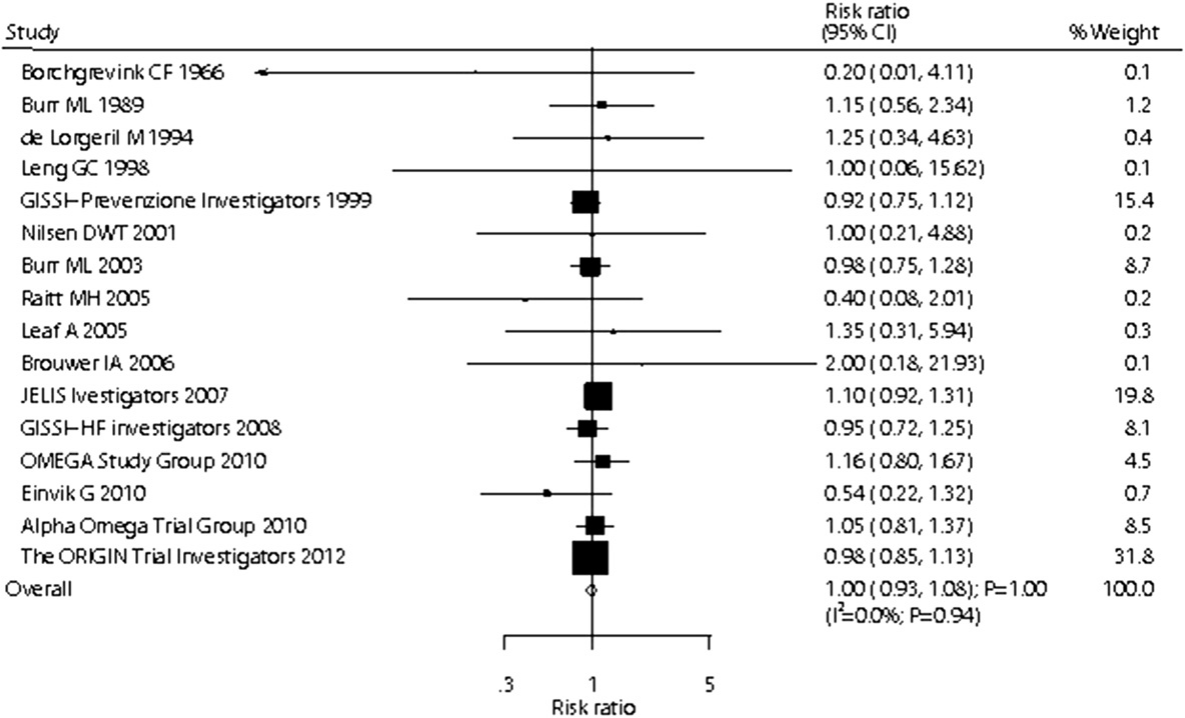
Effect of omega-3 fatty acid supplementation on the risk of nonvascular death.

Data for the effect of omega-3 fatty acid supplementation on nonvascular death included 66,204 individuals and 2,439 events of nonvascular death. There were no differences observed between participants receiving omega-3 fatty acid versus those receiving the placebo for nonvascular death (RR, 1.00; 95% CI: 0.93–1.08; P = 1.00, Figure 3), and no statistical heterogeneity was observed between trials (P = 0.94). After sequential exclusion of each trial from all pooled analysis, the results were not affected by exclusion of any specific trial. In a cumulative meta-analysis for nonvascular death (Additional file 4: Figure S3), the omega-3 fatty acid effect remained unchanged, i.e., not statistically significant.

**Figure 4:**
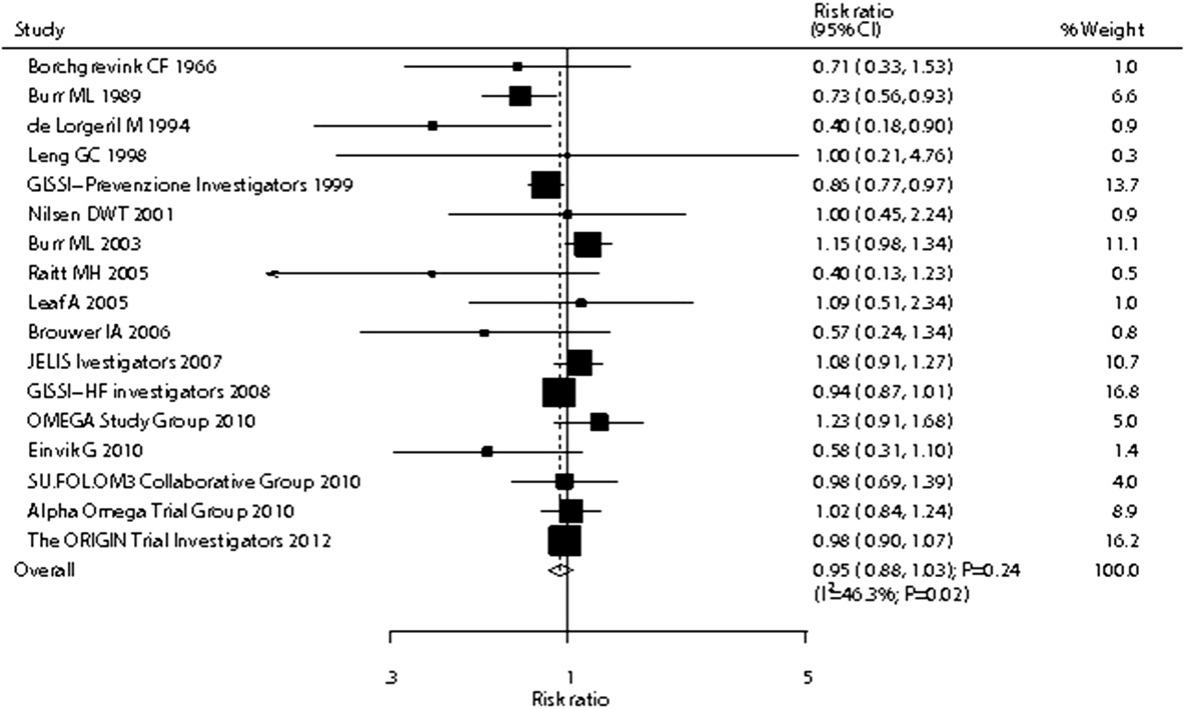
Effect of omega-3 fatty acid supplementation on the risk of total mortality.

Data for the effect of omega-3 fatty acid supplementation on total mortality included 68,705 individuals and reported 7,025 total mortality events. We noted that omega-3 fatty acid supplementation showed a 5% reduction in total mortality; however, there was no supporting evidence to show that omega-3 fatty acid protected against total mortality risk (RR, 0.95; 95% CI: 0.88–1.03; P = 0.24, Figure 4). Heterogeneity was observed in the magnitude of the effect across the trials (I^2^ = 46.3%; P = 0.02). However, after sequential exclusion of each trial from all pooled analysis, the conclusion was not affected by the exclusion of any specific trial. In a cumulative meta-analysis for total mortality (Additional file 5: Figure S4), the originally proposed significant omega-3 fatty acid effect was refuted by evidence accumulated up to 2003 and has continued to linger around a small effect and borderline statistical significance.

Heterogeneity testing for the analysis showed a P > 0.10 for cancer incidence and nonvascular death. We concluded that heterogeneity is not significant in the overall analysis, suggesting that most variation was attributable to chance alone. However, substantial heterogeneity was observed in the magnitude of the effect for total mortality across the trials. We, therefore, conducted a meta-regression [24] analysis including sample size, mean age, percent men, and duration of the follow-up. However, these variables did not seem to be important factors contributing to the association between omega-3 fatty acid supplementation and total mortality risk (sample size, P = 0.302; mean age, P = 0.391; percent men, P = 0.804; and duration of follow-up, P = 0.786).

Subgroup analyses were also conducted for cancer incidence, nonvascular death, and total mortality to evaluate the effect of omega-3 fatty acid supplementation in a specific population. We noted that omega-3 fatty acid supplementation was associated with a reduction in total mortality risk if the trials were published before 2000, the number of individuals in the study was less than 1000, the proportion of men in the study was more than 80%, or participants received alpha-linolenic acid. No other significant differences were identified in pre-defined factors between those who took omega-3 fatty acid or placebo. Furthermore, there was no other significant difference in the effects of omega-3 fatty acid between the 2 subgroups by factors that could affect the treatment effects (Table 2).

**Table 2:** Subgroup analysis for the effect of omega–3 fatty acid supplementation on cancer incidence, nonvascular death, and total mortality

**Outcomes**	**Group**	**Number of study**	**RR and 95% CI**	**P value**	**Heterogeneity (%)**	**P value for heterogeneity**	**P value for interaction test**
**Cancer incidence**	**Publication year**						
	Before 2000	5	1.07 (0.85–1.34)	0.56	0	0.87	0.789
	After 2000	6	1.11 (0.96–1.28)	0.15	0	0.93	
	**Number of patients**						
	>1000	5	1.10 (0.97–1.24)	0.12	0	0.98	0.878
	<1000	6	1.03 (0.45–2.38)	0.94	0	0.83	
	**Percent men (%)**						
	>80	7	1.06 (0.87–1.28)	0.58	0	0.98	0.608
	<80	4	1.13 (0.97–1.31)	0.13	0	0.72	
	**Mean age**						
	>64	1	5.00 (0.24–103.28)	0.30	–	–	0.328
	<64	10	1.10 (0.97–1.24)	0.13	0	1.00	
	**Intervention**						
	Alpha–linolenic acid	1	0.33 (0.01–8.09)	0.50	–	–	0.481
	Long–chain n–3 PUFA	10	1.10 (0.98–1.24)	0.12	0	0.99	
	**Primary or secondary prevention**						
	Primary	1	1.11 (0.93–1.33)	0.26	–	–	0.882
	Secondary	10	1.09 (0.93–1.28)	0.29	0	0.98	
	**Duration of the follow–up period (months)**						
	>36	4	1.10 (0.97–1.24)	0.14	0	0.98	0.883
	<36	7	1.16 (0.58–2.33)	0.68	0	0.88	
	**Jadad score**						
	>4	6	1.11 (0.96–1.30)	0.16	0	0.98	0.771
	<4	5	1.07 (0.88–1.30)	0.49	0	0.77	
**Nonvascular death**	**Publication year**						
	Before 2000	5	0.93 (0.77–1.13)	0.47	0	0.82	0.440
	After 2000	11	1.01 (0.93–1.10)	0.75	0	0.86	
	**Number of patients**						
	>1000	8	1.01 (0.93–1.09)	0.89	0	0.89	0.328
	<1000	8	0.77 (0.45–1.32)	0.34	0	0.80	
	**Percent men (%)**						
	>80	9	0.93 (0.80–1.08)	0.37	0	0.78	0.219
	<80	6	1.04 (0.94–1.14)	0.47	0	0.92	
	**Mean age**						
	>64	7	1.01 (0.86–1.19)	0.89	0	0.83	0.916
	<64	9	1.00 (0.91–1.09)	0.94	0	0.78	
	**Intervention**						
	Alpha–linolenic acid	3	0.95 (0.32–2.85)	0.93	0	0.54	0.927
	Long–chain n–3 PUFA	13	1.00 (0.93–1.08)	1.00	0	0.89	
	**Primary or secondary prevention**						
	Primary	3	1.01 (0.87–1.18)	0.87	31.6	0.23	0.754
	Secondary	13	0.98 (0.88–1.10)	0.78	0	0.97	
	**Duration of the follow–up period (months)**						
	>36	7	0.99 (0.92–1.08)	0.85	0	0.68	0.460
	<36	9	1.11 (0.83–1.49)	0.49	0	0.92	
	**Jadad score**						
	>4	8	1.02 (0.93–1.11)	0.71	0	0.62	0.427
	<4	8	0.95 (0.82–1.11)	0.54	0	0.97	
**Total mortality**	**Publication year**						
	Before 2000	5	0.79 (0.67–0.93)	0.005	19	0.30	0.009
	After 2000	12	1.00 (0.94–1.08)	0.90	29	0.16	
	**Number of patients**						
	>1000	9	0.98 (0.91–1.06)	0.57	57	0.02	0.014
	<1000	8	0.67 (0.50–0.90)	0.007	0	0.59	
	**Percent men (%)**						
	>80	9	0.80 (0.65–0.98)	0.03	64	0.005	0.033
	<80	7	1.01 (0.95–1.08)	0.71	0	0.84	
	**Mean age**						
	>64	7	0.96 (0.89–1.02)	0.20	0	0.44	0.543
	<64	10	0.92 (0.82–1.04)	0.18	62	0.004	
	**Intervention**						
	Alpha–linolenic acid	3	0.58 (0.35–0.98)	0.04	0	0.46	0.058
	Long–chain n–3 PUFA	14	0.96 (0.89–1.04)	0.36	47	0.03	
	**Primary or secondary prevention**						
	Primary	3	0.99 (0.86–1.14)	0.91	46	0.15	0.566
	Secondary	14	0.94 (0.84–1.04)	0.21	47	0.03	
	**Duration of the follow–up period (months)**						
	>36	8	0.98 (0.91–1.05)	0.54	47	0.06	0.157
	<36	9	0.80 (0.61–1.05)	0.11	43	0.08	
	**Jadad score**						
	>4	9	0.98 (0.91–1.06)	0.65	30	0.18	0.320
	<4	8	0.88 (0.72–1.07)	0.19	59	0.02	

A review of funnel plots could not rule out the potential for publication bias for lung cancer (Additional file 6: Figure S5). The Egger [26] and Begg test [27] results showed no evidence of publication bias for cancer incidence (Egger: P = 0.663; Begg: P = 0.876), nonvascular death (Egger: P = 0.519; Begg: P = 0.620), and total mortality (Egger: P = 0.255; Begg: P = 0.174).

## Discussion

Several previous observational studies [1-3] have suggested that omega-3 fatty acid supplementation has a marked effect on cancer incidence. Kato and colleague [1] performed a prospective study of omega-3 fatty acid (fish or shellfish diet) and colorectal cancer in New York and Florida. This prospective study included 14724 individuals and found a 51% reduction in the risk of colorectal cancer with omega-3 fatty acid dietary consumption. Furthermore, Takezaki et al. [2] concluded that frequent fresh fish consumption, irrespective of the cooking method, might reduce the risk of lung cancer. However, the effect of omega-3 fatty acid supplementation in reducing the risk of incident cancer has not been confirmed by randomized controlled trials and meta-analysis.

Previous systematic review [36] have evaluated the impact of omega-3 fatty acid supplementation on cancer incidence on the basis of observational studies. These studies encompass a large body of literature spanning numerous cohorts, from many countries, with different demographic characteristics and do not provide any evidence to support a significant association between omega-3 fatty acid and cancer incidence. Our study was based solely on randomized controlled trials and explored all possible correlations between omega-3 fatty acid supplementation and the outcomes of cancer incidence, nonvascular death, and total mortality. This large quantitative study included 68,954 individuals from 19 trials with a broad range of baseline characteristics. The results of our meta-analysis suggest that omega-3 fatty acid supplementation has no effect on the incidence of cancer, nonvascular death, and total mortality.

Our main findings are inconsistent with the findings of previous epidemiologic research [1-3], and concluded that omega-3 fatty acid supplementation had no significant benefit or adverse effect on the risk of cancer incidence, nonvascular death or total mortality. The reason for this could be that observational studies may overestimate the effect of omega-3 supplementation.

There was no significant difference between omega-3 fatty acid supplementation and the placebo for the relative risk of cancer incidence. Omega-3 fatty acids do not seem to affect a mechanism of cancer development that is common across the different types of cancers evaluated in this study. Previous observational studies [1-3] suggested that risk reductions were observed for colorectal, lung, and prostate cancer. However, data on any specific cancer type were unavailable in this study; therefore, we did not identify the association between any specific type of cancer incidence and omega-3 fatty acid supplementation. According to our study, omega-3 fatty acid supplementation resulted in a 10% increase in the risk of cancer incidence, but this difference was not statistically significant. The possible reasons for this lack of significant effect are as follows: (1) the use of background omega-3 fatty acid supplementation might have impaired our ability to identify a treatment effect, and (2) trials included were designed to evaluate the effects of omega-3 fatty acid supplementation on cardiovascular outcomes, but not cancer-related outcomes, these results were derived from very few cases and should be regarded as preliminary results. Furthermore, high intake of omega-3 fatty acid might stimulate carcinogenesis by increasing oxidative DNA damage [37]. Finally, studies with an open, controlled design may have introduced behavioral differences which may have had an impact on the development of cancer. Therefore, although omega-3 fatty acid supplementation may have direct effects on cancer incidence, these effects may be balanced. Furthermore, subgroup analysis also supports that omega-3 fatty acid supplementation does not affect cancer incidence.

Omega-3 fatty acid supplementation might play an important role in reducing the risk of total mortality, due to the improved effects on cardiac death [38]. Our study suggests that omega-3 fatty acid produced a 5% reduction in total mortality; however, this reduction was not statistically significant. The reason for this absence of statistical difference could be that omega-3 fatty acids have no effect on the risk of nonvascular death. Hence, this effect may be reduced by nonvascular death. Subgroup analysis was also conducted on total mortal risk based on pre-defined factors. We noted that omega-3 fatty acid supplementation reduced the risk of total mortality only when we included trials published before 2000, the sample size was less than 1000, the proportion of men in the population was more than 80%, or participants received alpha-linolenic acid. The reason for these findings could be that several factors might affect the efficacy of treatment, which happened to occur more frequently in male patients, such as smoking status.

The limitations of our study are as follows: (1) relatively few events of cancer incidence were reported, which always contributed to broad confidence intervals, and restricted us from obtaining an intrinsic effect; (2) different types of supplements might provide a biased view of the study question; (3) data on any specific type of cancer were unavailable, due to which we could not identify the association between any specific type of cancer incidence and omega-3 fatty acid supplementation; and (4) since inherent assumptions are made for any meta-analysis, the analysis used pooled data, and individual patient data was not available, which restricted us from performing a more detailed relevant analysis and obtaining more comprehensive results.

## Conclusions

The findings of this study suggest that omega-3 fatty acid supplementation has no significant effects on cancer incidence, nonvascular death, or total mortality. Future studies should focus on healthy individuals to analyze the primary prevention of cancer incidence. We suggest that ongoing trials should be improved in the following ways: (1) any specific type of cancer and total cancer incidents should be recorded and reported normatively, and it should be evaluated in any future trial, and (2) the role of intervention duration and dosage of supplementation should be taken into consideration before evaluating clinical outcomes.

### Ethics

An ethics statement was not required for this work.

## Competing interests

The authors declared that they have no competing interests.

## Authors’ contributions

Conceived and designed the experiments: Z-YH. Performed the experiments: Z-YH, G-HF, H-AJ and Z-YF. Analyzed the data: Z-YH, and Z-YF. Contributed reagents/materials/analysis tools: Z-YH. Wrote the paper: Z-YH. All authors read and approved the final manuscript.

## Pre-publication history

The pre-publication history for this paper can be accessed here:

http://www.biomedcentral.com/1471-2458/14/204/prepub

## Supplementary Material

Additional file 1: Table S1 PRISMA Checklist. Click here for file

Additional file 2: Figure S1 PRISMA Flowchart. Click here for file

Additional file 3: Figure S2 Cumulative meta-analysis of the omega-3 fatty acid supplements for cancer incidence. Click here for file

Additional file 4: Figure S3 Cumulative meta-analysis of the omega-3 fatty acid supplements for nonvascular death. Click here for file

Additional file 5: Figure S4 Cumulative meta-analysis of the omega-3 fatty acid supplements for total mortality. Click here for file

Additional file 6: Figure S5 Funnel plot for cancer incidence, nonvascular death, and total mortality. Click here for file
