# Supplementary figures and images for: Effect of omega-3 fatty acid supplementation on cancer incidence, non-vascular death, and total mortality: a meta-analysis of randomized controlled trials
Source: BMC Public Health. 2014 Feb 26;14:204. doi: 10.1186/1471-2458-14-204 (PMC3938028; doi:10.1186/1471-2458-14-204)

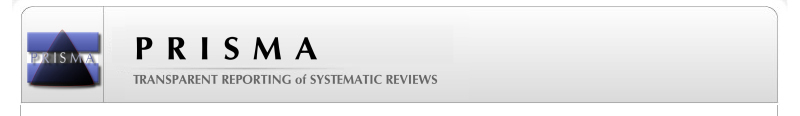
**PRISMA 2009 Flow Diagram**

**Screening**

**Included**

**Eligibility**

**Identification**

Supplement: Additional file 2: Figure S1 — PRISMA Flowchart. [file 1471-2458-14-204-S2.doc]
